# The Importance of Medication Errors Reporting in Improving the Quality of Clinical Care Services

**DOI:** 10.5539/gjhs.v8n8p243

**Published:** 2015-12-17

**Authors:** Nesreen Mohamed Kamal Elden, Amira Ismail

**Affiliations:** 1 Department of Public Health and Community medicine, Faculty of Medicine, Cairo University, Egypt; 2 Dar El Shefaa Hospital, Ministry of Health and Population, Egypt

**Keywords:** medication errors, reporting, patient safety

## Abstract

**Introduction:**

Medication errors have significant implications on patient safety. Error detection through an active management and effective reporting system discloses medication errors and encourages safe practices.

**Objectives:**

To improve patient safety through determining and reducing the major causes of medication errors (MEs), after applying tailored preventive strategies.

**Methodology:**

A pre-test, post-test study was conducted on all inpatients at a 177 bed hospital where all medication procedures in each ward were monitored by a clinical pharmacist. The patient files were reviewed, as well. Error reports were submitted to a hospital multidisciplinary committee to identify major causes of errors. Accordingly, corrective interventions that consisted of targeted training programs for nurses and physicians were conducted.

**Results:**

Medication errors were higher during ordering/prescription stage (38.1%), followed by administration phase (20.9%). About 45% of errors reached the patients: 43.5% were harmless and 1.4% harmful. 7.7% were potential errors and more than 47% could be prevented. After the intervention, error rates decreased from (6.7%) to (3.6%) (P≤0.001).

**Conclusion:**

The role of a ward based clinical pharmacist with a hospital multidisciplinary committee was effective in recognizing, designing and implementing tailored interventions for reduction of medication errors. A systematic approach is urgently needed to decrease organizational susceptibility to errors, through providing required resources to monitor, analyze and implement effective interventions.

## 1. Background

Medication error is defined as any preventable event that may cause or lead to inappropriate medication use or patient harm while the medication is under the control of the health care professional, patient, or consumer (National Coordinating Council for Medical Reporting and Prevention, (NCCMERP), 2012)

These errors may occur during any phase of the drug delivery process from prescription to drug administration and at anywhere medications are administered (Fortescue et al., 2003). Those various phases are not exclusive because the origins of errors are frequently multiple.

Medication errors (MEs) are among the most common medical errors, harming about 1.5 million people every year. Medication errors are main contributors to adverse events to hospitalized patients (Bates et al., 1999; Bates, Boyle, Vanda Vliet, Schneider, & Leape, 1995). In addition to weakening the patients’ confidence in medical services, MEs also impose substantial costs between US$ 6 billion and US$ 29 billion per year (WHO, 2014).

MEs prolong hospital stays by 2 days and increase the costs by $2,000-$2,500 per patient (Bates et al., 1995; Classen, Pestotnik, Evans, Lloyd, & Burke, 1997; Vasin, Zamamin, & Hatam, 2014).

Quality has been a major focus of concern in health care for numerous decades. The Joint Commission on Accreditation of Health Care Organization reemphasized the importance of the analysis of error reports to prevent future errors with the implementation of additional patient safety standards that address the development of a culture of safety (Joint Commission Accreditation of Health Care Organization (JCAHO), 2001). Error reporting helps to understand why errors occur, to prioritize opportunities for error prevention and to generate long term improvements in patient safety (Claudia, Sharon, DeVSP, Merrell, & Gail, 2002).

Although several studies have demonstrated that specific interventions in the medication orders and processing might reduce the risk of error (Kaushel et al., 2001), many hospitals have no system for recording medical errors which are thus under reported across Health Care organizations.

Voluntary reporting by health care providers depends on their awareness, hence many errors remain frequently unreported (WHO, 2014). The percent of under reporting of adverse events is estimated to range from 50% to 60% annually. Despite their high incidence, medication errors reporting in the medical care practice is usually done in an informal manner (Barach & Small, 2000). Errors are discussed verbally at morbidity or mortality meetings. Without formal written reports, patient safety improvement opportunities will be very limited (Claudia et al., 2002**)**.

Errors should be identified through an active management and effective reporting system, so they could be removed before they can reach or cause harm to patients.

The study aims to improve patient safety services through the following objectives:

1) Determine the baseline rates of medication errors in the hospital;
2) Recognize the major types of medication error;
3) Reduce risks of medication errors through application of prevention strategies.

## 2. Methodology

### 2.1 Study Site

The study was conducted at the internal Patients wards of Dar El Shefaa Hospital, which is a health care organization, under the supervision of the Specialized Medical Centers Department of Ministry of Health (MOH). The hospital is formed of 5 floors, with 177 beds. The hospital provides clinical services in different specialties: Internal Medicine, General Surgery, Gynecology and Obstetrics, Ophthalmology, Neurology, Neurosurgery, Chest, Cardiothoracic surgery, renal … In addition to laboratory and radiological services.

### 2.2 Study Design

The study is a health system research Pretest/post test intervention study. A three-phase study was performed consisting of a pretest phase and a subsequent post test phase separated by a corrective intervention phase.

The details of the three phases were as follows:

**a- The pretest phase:**

During the pretest phase, a multidisciplinary hospital committee was formed from the following staff members:

- A member from quality department.
- A member from training center department.
- A Clinical Pharmacist.
- A member of Physicians.
- A member of nursing staff.

The committee reinforced medication errors reporting policy in the hospital internal wards, after approval of the hospital administrative authorities. The medication errors’ report includeed all errors related to medication (appendix). Voluntary reports from physicians and nurses were collected on standardized forms. In addition, errors in the hospital wards were monitored daily by clinical pharmacists, from June 2012 till November 2012. The clinical pharmacists’ monitored drug handling stages and reviewed the files of the patients. They documented all procedures, and assured accordance with the predefined error stages. The monitors intervened, according to their own judgment, if there were potential or preventable errors. All error reports were submitted to the committee to identify and eliminate common causes of errors.

The committee reviewed all submitted reports. The incidence and the types of medication errors, within the hospital’s wards, were identified.

**b- The intervention phase:**

An analysis of the collected reports was conducted. A quantitative analysis was performed according to medication stages to prioritize risks related to prescription, dispensing, preparation, administration and monitoring stages. Identification of MEs that caused or had the potential to cause harm was done through the quantitative analysis of patient outcome. In addition, a root cause analysis (qualitative analysis) was conducted for better understanding of contributing causes and root factors. Accordingly, a corrective intervention that consisted of a targeted training program for nurses and physicians was developed and conducted during the following two months. The training for nurses and physicians aimed to improve their awareness of the importance of the medical sheet and of the commonly identified causes of medication errors. The role of error reporting and effective communication to promote patient safety was explained. 53% of physicians (n=32) and 71% of nurses (n=64) received one day training session.

**C- The post test phase:**

The monitoring was continued for the next five months after the training. The incidence of the medication errors were then recalculated and compared to the pretest results.

### 2.3 Sampling

Assuming a prevalence of at least one error in 50% of the administrative procedures of drugs administered before intervention and a reduction of about 50%, that is, an error prevalence of not more than 25% of the administrative procedures after the intervention in an independent patient group. At least, fifty seven drug administration tasks per group were needed to detect significant prevalence differences (α=0.05; 1-β=0.80). All patients admitted to the inpatient wards in the Dar El Shefaa Hospital, during baseline and post intervention phases, were included.

### 2.4 Data Collection

Voluntary reporting from physicians and nurses during the study period. In addition, the clinical pharmacist, in each ward, monitored drug handling stages and reviewed the patient files. Data collected, for each incident included: date, time, the person responsible with a full description of the incident.

### 2.5 Data Management

- **Outcome variable**
  Medication errors: were defined as errors in drug ordering or prescription, dispensing, preparing the medicine, administering or monitoring (Appendix).
  The medication process is the term used to describe the process of delivering medications to patients. It consisted of five stages:
  **Stage 1: -** Prescription the medicine.
  **Stage 2: -** Dispensing the medicine.
  **Stage 3: -** Preparing the medicine for administration.
  **Stage 4: -** Administering the dose using the appropriate route and method.
  **Stage 5: -** Monitoring the effect of the medicine to the patient.
    The (NCCMERP, 2012) taxonomy was used as a guide to categorize MEs, according to patient outcome into:
- **Potential errors:** Circumstances or events that have the capacity to cause the error.
- **Prevented errors:** Errors that didn’t reach the patient.
- **Harmless errors:** Errors that reached the patient and didn’t cause harm.
- **Harmful errors:** Errors that reached the patient and caused harm.

Medication errors were calculated depending on the number of doses that have been taken by the patients, who were included in the study, throughout their stay at the hospital.

The error rate was calculated for 100 doses, according to the following equation (Total number of errors/Total number of doses * 100).

Pre coded data was entered on Microsoft Office Excel Program for Windows, 2007. Data was then transferred to the Statistical Package of Social Science, version17 (SPSS.17). The P value ≤ 0.05 was used as the cutoff level for statistical significance. Chi-square was used to detect associations between categorical variables.

### 2.6 Ethical Consideration

The researcher described the objectives of the study to the hospital administrative authorities and got their verbal approval to conduct the research in the facility. All physicians, nurses and pharmacists, involved in drug administration were invited to participate in the study.

## 3. Results

1467 medication errors were reported during the pretest phase for a total of 21843 doses (6.7%) versus 1380 errors from 38104 observed doses (3.6%) during the post test phase, (P ≤ 0.001).

Table 1 showed that about 45% of errors reached the patients: 43.5% were harmless and 1.4% of errors caused harm. More than 47% of the errors could be prevented if adequate measures were applied on time.

**Table 1:** Percent distribution of medication errors, according to their harm capacity

Errors Harm Capacity	N (%)
**Potential Errors**	**113 (7.7%)**
**Prevented Errors**	**695 (47.4%)**
**Harmless Errors**	**638 (43.5%)**
**Harmful Errors**	**21 (1.4%)**
**Total**	**1467(100%)**

**Figure 1:**
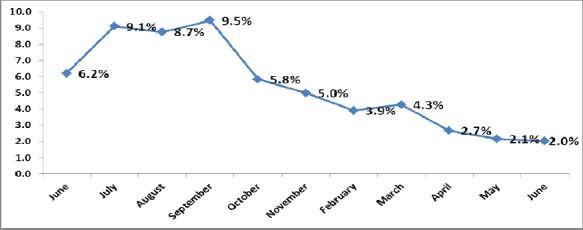
Percent distribution of medication errors during the study period

Figure 1 displayed the percent of medication errors from total observed doses during the study period: Medication errors decreased from (6.2%) of the total doses at the beginning of the study period (June 2012) to (2%) by the end of the study period (June 2013).

Table 2 illustrated the percent distribution of medication errors, according to the drug handling stage. A total of 1467 errors occurred during pretest phase (six months) versus 1380 errors during the following five months after the intervention. Medication errors were higher during ordering/prescription phase (38.1%), followed by administration phase (20.9%). Medication errors decreased, after the intervention with all drug handling stages except during the monitoring phase: (25.9%) in the post test versus (18.3%) during pretest phases, (P=0.001). MEs during dispensing phase decreased significantly from (10.4%) to (7.0%), (P=0.002).

**Table 2:** Percent distribution of medication errors by stages during pretest and post test phase

Types of errors	Pretest	Post test	P

	N (%)	N (%)	
**Ordering/Prescribing**	**559 (38.1%)**	**490 (35.5%)**	**0.1**
**Dispensing**	**153 (10.4%)**	**96 (7.0%)**	0.002*
**Preparation**	**181 (12.3%)**	**161 (11.7%)**	**0.7**
**Administration**	**307 (20.9%)**	**275 (19.9%)**	**0.5**
**Monitoring**	**267 (18.3%)**	**358 (25.9%)**	0.001*
**Total**	**1467 (100%)**	**1380 (100%)**	

* P≤ 0.05

**Table 3:** Percent distribution of medication errors by responsible person

Person Responsible	Pretest	Post test

	N (%)	N (%)
**Doctors**	**836(57.0%)**	**729 (52.8%)**
**Pharmacist**	**335 (22.8%)**	**362 (26.2%)**
**Nurses**	**296 (20.2%)**	**289 (21.0%)**
**Total**	**1467 (100%)**	**1380 (100%)**
**P Value**	**0.06**	

The training program was successful in decreasing the percent of errors caused by doctors from (57%) to (52.8%). On the other hand, the errors caused by the pharmacists and the nurses increased insignificantly from (22.8%) to (26.2%) and from (20.2%) to (21%), respectively after the intervention. (P=0.06).

## 4. Discussion

The goal of the drug therapy is the achievement of the best therapeutic outcomes and the improvement of the patient’s quality of life. Unfortunately, there are hazards associated with the therapeutic use of drugs, including adverse drug reactions and medication errors (Helper & Stand, 1990).

Medication errors have significant implications on patient safety. These errors occur at all stages in medication use: ordering, prescription, dispensing, and administration. Error detection discloses those errors and thus, encourages a safe culture (Montesi & Lechi, 2009).

In an earlier study at two teaching hospitals, 616 MEs were found out of 10778 observed medication doses at a rate of (5.7%) (Kaushel et al., 2001). Among 16,938 medication orders in a study on pediatric inpatients at a large academic community hospital, there were 865 medication errors noted, a rate of 5.2 per 100 orders (Wang et al., 2007). In the current study, medication errors constitute (6.7%) of all the administrations: 1467 errors out of 21843observations.

In an earlier Egyptian study, 1107 of the medication orders (78.1%) had at least one prescription error (Alagha, Badary, Ibrahim, & Sabry, 2011). In a descriptive analysis of medication errors reported to the Egyptian national online reporting system, out of the 12000 valid reports that were analyzed, prescription errors were the most common type of MEs (54%), followed by monitoring (25%) and administration errors (16%) (Shehata, Sabry, & Elmelegy, 2015).

In the current study: The most common stage for medication errors was during the ordering and prescription stage (38.1%), followed by the administration stage, (20.9%). Errors during monitoring, preparation and dispensing were: (18.3%), (12.3%) and (10.4%), respectively.

Published research indicates that not all MEs cause harm (WHO, 2014). Several approaches that are used to assess errors preventability depend mainly on the researcher’s own judgement. (Ferner & Anorson, 2006)

In the Egyptian study (Shehata et al., 2015), (25%) and (11%) of errors were potential and prevented errors versus (7.7%) and (47.4%) in this study, respectively. About 45% of errors in Dar El Shefaa, during the baseline measurement, affected patients: 43.5% were harmless and 1.4% was harmful versus 51% harmless and 13% harmful errors reported to the Egyptian national online reporting system, from June to December 2014.

There are multiple tools for error detection given the available resources. Medication errors are detected by voluntary reporting, direct observation, and chart review. Organizations need to establish systems for prevention of medication errors through analyzing the cause of errors to identify opportunities for quality improvement and system changes (Morimoto, Seger, Hsieh, & Bates, 2004).

Each tool has its specific advantages: and its limitations: e.g. direct observation is used for detection of dispensing and administrative errors more than prescription and monitoring errors. Chart review is a reliable method for detection of prescription errors more than detection of administrative errors (Morimoto et al., 2004; WHO, 2014). For that, in the current study errors during drug prescription, dispensing, preparation and administration phases, decreased from: (38.1%) to (35.5%); (10.4%) to (7.0%), (12.3%) to (11.7%) and from (20.9%) to (19.9%) respectively. On the other hand, monitoring errors increased significantly from (18.3%) to (25.9%).

In a study to identify and prioritize effective strategies to reduce medication errors: 81% of the physicians suggested to activate the role of a ward based clinical pharmacists (Fortescue et al., 2003). Clinical pharmacists are trained in therapeutics and provide comprehensive drug management to patients and providers. Accordingly, clinical pharmacists are capable of managing medication therapy in a patient care setting, in collaboration with other health care professional (Peter, Kaboli, Angel, Hoth, & McClimer, 2006)

The pharmacist’s role is to ensure that all patients reach the optimum benefit from medications. Through a system-oriented approach, the pharmacist should lead coordinated, multidisciplinary efforts to prevent and detect drug-related problems that could cause harm (Helper et al., 1990). The pharmacist should participate in appropriate organizational committees and work with physicians, nurses, and other staff to ensure that medication processes are safe (Lesar et al., 1990). Hence, in the current study, the pharmacist, in each ward, monitored all stages of medication, from the drug’s ordering to the administration of the drug. They reviewed patient files and prepared errors’ report. A multidisciplinary committee reviewed all submitted reports. Accordingly, the training program was developed and conducted during the intervention phase. The intervention succeeded to decrease the error rate by 46.3%.

The medication errors decreased progressively during the study period (3.6%) in the post test period versus (6.7%) of all observed doses during the pretest phase.

In a study to examine the impact of clinical pharmacist intervention in preventing harm from medication errors in 2 children’s hospitals during 6-month period, the error rates before and after clinical pharmacist intervention were 4.9 and 4.5 errors per 1000 medication orders, respectively (Folli, Poole, Benitz, & Russo, 1987).

Utilizing a continuous quality improvement approach, a 2 year prospective cohort study used an adverse incident reporting scheme and a multidisciplinary committee to analyze medication error reports. Consequently, changes in policy and practice were implemented to reduce the errors. During the second year, the incidence of all reported errors fell (Wilson et al., 1998).

The incidence of medication errors decreased significantly, from 49% to 31% after an educational intervention in medication preparation and administration in a tertiary Neonatal Intensive Care Unit (Chedoe, Molendijk, Hospes, Van den Heuvel, & Taxis, 2012).

In a study of medication errors among neonatal and pediatric inpatients, including the impact of a series of interventions to reduce errors: before the interventions, the medication error rate was 11.4% compared with 7.3% after the interventions (Otero, Leyton, Mariami & Gernadas, et al., 2008).

The introduction of tutorial prescribing routine in a pediatric unit at a district general hospital in the United Kingdom decreased prescription errors by 46% (Davey, Britland, & Naylor, 2008).

In the current study, ordering and prescription errors was the most common cause of MEs (38.1%) during the baseline measurement and decreased to (35.5%) following the intervention.

Physicians were responsible for 72% of errors in a study of medication errors in pediatric practice (Wilson et al., 1998). Physicians of Dar El Shefaa were responsible for 57% of MEs that decreased to 52.8% of errors after staff education. On the other hand the percent of errors caused by nurses and pharmacists increased insignificantly during the same period.

The study highlights the importance of error reports as sources of information for the generation of preventive strategies aimed toward medication error reduction. Reports were analyzed quantitatively and qualitatively for identification and prioritization of error medication stages, their effect on the patient and their root causes. Hence corrective actions targeted priority areas and root causes to prevent recurrence. The study emphasized both the need and effectiveness of quality-improvement programs that focus on educating the staff about the medication errors and the importance of reporting. Monitoring by clinical pharmacists proved to be an effective method for error detection. The strategies explored in this study can assist any organization in decreasing rates of medication errors and in patient safety enhancement efforts. Due to the high risk of MEs among pediatrics and intensive care units, many earlier studies described MEs among those populations. In this study, the risk of MEs among adults in the inpatient wards was investigated extensively.

The study had some limitations:

The differences of definitions and of error detection methods limit the comparison among different studies (Vasin et al., 2014). Organizations involved in reporting MEs use different terms and definitions. For an effective collaboration, there is a need to a common” language” (Yu, Nation, & Dooley, 2005; WHO, 2014). The intervention was conducted over a limited duration (two monthes) which may have affected the findings.

In addition, using the direct observation method for determining MEs was found to cause sometimes behavioral changes in the observed as the health care providers were aware of the study; the Hawthorne effect could have affected both the occurrence and detection of errors (Dean & Barber, 2001). It is also possible for the observer to fail to record certain MEs. The incidence of errors could have been reduced as the study progressed because corrective actions were taken as soon as serious practice problems were identified.

## 5. Conclusion and Recommendations

Error reporting and cause analysis are important tools to identify the major causes of medication errors. Medication error reporting systems should be improved by removing barriers and by clarifying the importance and the role of health care professionals. The role of ward-based clinical pharmacists with a hospital multidisciplinary committee in recognizing, designing tailored preventive strategies and minimizing medication error was effective and should be supported. Educational and training programs on drug therapy are required for medical/paramedical students, drug prescribers (doctors) and nurses (administrating drugs) to reduce drug errors and to improve patient safety. A systematic approach is urgently needed to decrease organizational susceptibility to error, through providing required resources to monitor, analyze cause of errors and implement preventive strategies to reduce them. A proper functioning national standardized system for MEs detection and reporting using a unified terminology all over the country is necessary to allow for better knowledge sharing and practice change.

## Appendix:

**Appendix A**

**Table A1:** List of medication errors required to be reported in Dar El Shefaa hospital

[O] Ordering and Prescribing	[D] Dispensing	[P] Preparation	[A] Administration	[M] Monitoring
**1**incomplete patient data *(age- wt- height- diagnosis)* **2**illegible handwriting **3**abbreviations **4**duplication **5**incomplete or wrong drug information *(name - strength - dose - route - freq - days - date - sign - directions)* **6**drug Discontinuation error **7**drug Modification error **8**drug not written in right place **9**med. Sheet not renewed **10**med. Sheet not matched with dispensing order **11**drugs with patient but not written in med. Sheet or drug history **12**others	**1**wrong dispensing *(patient - drug - dose-dosage form-quantity)* **2**drug not dispensed *(forgotten - not available - incomplete order)* **3**dispensing of drug that is discontinued or exceeded days of therapy **4**incomplete labeling **5**poor packaging **6**delayed dispensing **7**unreturned drugs **8**others	**1**wrong patient **2**error in dilution and/or compatibility **3**no Hypersensitivity test **4**unlabeled drug **5**delayed preparation **6**drug not prepared **7**drug prepared in wrong time **8**non-adherence to inf. Control standards **9**missed Pharmacist signature **10**others	**1**wrong patient **2**wrong time **3**omitted administration **4**no signature **5**wrong or false signature **6**non - adherence to directions of administration *(hypersensitivity test - infusion rate- route)* **7**non - adherence to inf. control standards **8**medication left beside patient **9**others	**1**drug selection **2**dose *(overdose-sub theraputic-adjustment)* **3**drug interaction **4**adverse drug reaction **5**follow up of lab results or cultures **6**other
